# Does health literacy mediate the relationship between socioeconomic status and health related outcomes in the Belgian adult population?

**DOI:** 10.1186/s12889-024-18676-7

**Published:** 2024-04-27

**Authors:** Finaba Berete, Lydia Gisle, Stefaan Demarest, Rana Charafeddine, Olivier Bruyère, Stephan Van den Broucke, Johan Van der Heyden

**Affiliations:** 1 Department of Epidemiology and Public Health, Sciensano, Juliette Wytsmanstraat 14, Brussels, 1050 Belgium; 2 Department of Public Health, Epidemiology and Health Economics, University of Liège, Liège, Belgium; 3 WHO Collaborating Centre for Public Health Aspects of Musculoskeletal Health and Ageing Research Unit in Public Health, Epidemiology and Health Economics, University of Liege, Liège, Belgium; 4 Research Institute for Psychological Sciences, Louvain-la-Neuve, UCLouvain, Belgium

**Keywords:** Health disparities, Health literacy, Mediation analysis, Socioeconomic status

## Abstract

**Background:**

Health literacy (HL) has been put forward as a potential mediator through which socioeconomic status (SES) affects health. This study explores whether HL mediates the relation between SES and a selection of health or health-related outcomes.

**Methods:**

Data from the participants of the Belgian health interview survey 2018 aged 18 years or older were individually linked with data from the Belgian compulsory health insurance (*n* = 8080). HL was assessed with the HLS-EU-Q6. Mediation analyses were performed with health behaviour (physical activity, diet, alcohol and tobacco consumption), health status (perceived health status, mental health status), use of medicine (purchase of antibiotics), and use of preventive care (preventive dental care, influenza vaccination, breast cancer screening) as dependent outcome variables, educational attainment and income as independent variables of interest, age and sex as potential confounders and HL as mediating variable.

**Results:**

The study showed that unhealthy behaviours (except alcohol consumption), poorer health status, higher use of medicine and lower use of preventive care (except flu vaccination) were associated with low SES (i.e., low education and low income) and with insufficient HL. HL partially mediated the relationship between education and health behaviour, perceived health status and mental health status, accounting for 3.8–16.0% of the total effect. HL also constituted a pathway by which income influences health behaviour, perceived health status, mental health status and preventive dental care, with the mediation effects accounting for 2.1–10.8% of the total effect.

**Conclusions:**

Although the influence of HL in the pathway is limited, our findings suggest that strategies for improving various health-related outcomes among low SES groups should include initiatives to enhance HL in these population groups. Further research is needed to confirm our results and to better explore the mediating effects of HL.

**Supplementary Information:**

The online version contains supplementary material available at 10.1186/s12889-024-18676-7.

## Introduction

There is strong evidence that socioeconomic status (SES) is an important determinant of health disparities between population groups, with low SES being associated to poorer health conditions and less healthy behaviours [1–3]. Several factors and mechanisms have been proposed to explain the chain of events linking SES to health outcomes [2], including material circumstances (like living and working conditions), behavioural factors, social cohesion and social capital and lack of social support, as well as psychological factors like stress, social comparison, less coping resources and skills. However, the pathway through which SES exerts its effect on health has not yet been fully clarified [4].

Health literacy (HL) has been hypothesized as a potential mediator through which SES affects health [5–10]. According to the European Health Literacy Survey (HLS-EU) Consortium and the Health promotion glossary 2021, health literacy “is linked to literacy and entails a person’s knowledge, motivation and competences to access, understand, appraise, and apply health information in order to make judgments and take decisions in everyday life concerning healthcare, disease prevention and health promotion to maintain or improve quality of life during the life course” [11, 12]. The mediating effect of HL is assumed to be especially important for behaviours for which individual judgement and decision making are necessary, such as physical activity and diet [13] or self-rated health status [8, 9, 14, 15]. However, other factors beyond individual judgement and decision-making, such as political, structural, geographic, and historical forces are also of importance [16]. HL is an important factor for assessing public and personal health outcomes. A number of studies showed associations between low levels of HL and poorer health conditions [11, 12], more frequent use of health services, longer hospitalisations [11, 17] and higher mortality [12, 18]. Moreover, low level of HL has been associated with unhealthy behaviours, such as smoking [19, 20], low physical activity [20, 21] and less use of preventive services [12, 19]. On the other hand, HL has been associated with socioeconomic indicators such as educational attainment, income [9], material and social wealth or deprivation, unemployment status, occupation, as well as the sociodemographic profile (sex, age) of individuals [22]. In view of this, the World Health Organisation considers HL as an important determinant of health, influenced by socioeconomic and cultural characteristics of the population, and by the degree of complexity of the health systems [23]. As such, HL can be taken into account in efforts to reduce health disparities. Indeed, if HL is an important mediator in explaining socioeconomic (SE) health differences, actions to improve HL in low SE groups could reduce disparities [15].

In Belgium, equity in the use of healthcare resources is an important concern. However, empirical research investigating the contribution of HL in the relationship between SES and health remains scarce. To date, studies that have examined the mediating effects of HL have often failed to use a comprehensive questionnaire to measure HL [9, 14, 22], were carried out on a non-representative sample of the population [9], have had limited sample size (around 400 individuals) [24], have been limited to one or two specific health outcomes [9, 14] or did not perform mediation analysis, and only assessed the associations between HL, SES and health outcomes [8, 25]. This study aims to fill this gap. Using the linkage between two population-based data sources, it explores the mediation effect of HL in the association between SES and a selection of health outcomes classified into four domains: (1) health behaviours (physical activity, diet, alcohol and tobacco consumption), (2) health status (perceived health status, mental health), (3) use of medicine (purchase of antibiotics), and (4) use of preventive care (preventive dental care, influenza vaccination, breast cancer screening). These factors have been selected because a mediation effect of HL can be expected, given that each of them requires individual judgement and decision-making. More specifically, the hypothesis is that people with insufficient or limited HL have lower understanding of health promotion and intervention programmes and poorer management of their health problems because the system is not well devised to care for individuals with different competence and literacy levels, resulting in poorer health status.

The purpose of the present study is to determine whether HL mediates the associations between education and income (SES) and the above-mentioned health related outcomes. More specifically, the objectives are as follows:

1) to explore the association between SES and HL.

2) to examine the association between SES and the selected health related outcomes.

3) to examine the association between HL and the selected health related outcomes.

4) to investigate the mediation effects of HL in the relationship between SES and the selected health related outcomes.

Educational attainment and income are both explored as independent variables as a previous study has shown that the relationship between HL and income is independent of educational attainment [25].

## Methods

### Data and study population

The participants of this study were involved in the Belgian Health interview Survey (BHIS) 2018. The BHIS is a national, cross-sectional household survey conducted every 5 years since 1997 by Sciensano, the Belgian Public Health Institute, among a representative sample of Belgian residents. Participants are selected from the national population register, using a multistage, stratified-sampling design. For the 2018 edition of the BHIS, the participation rate of the survey at a household level was 57.5%. Information was collected on health status, health behaviour, HL, health care consumption, use of medicines and sociodemographic characteristics through a face to face interview and a paper and pencil questionnaire for the more sensitive questions. Detailed methodology of the survey can be found in Demarest et al. (2013) [26].

The BHIS data were individually linked to the Belgian Compulsory Health Insurance (BCHI) data using the unique national register number (HISlink 2018). The BCHI data contain exhaustive and detailed information on the reimbursed health expenses of over 99% of the total population. The database also includes a limited amount of sociodemographic information. The BCHI data were provided by the Intermutualistic Agency (IMA). IMA is a joint venture of the seven national health funds and collects and manages all data on healthcare expenditures as well as prescription information on reimbursed medicines (Pharmanet data) [27]. Pharmanet records all data on reimbursed medication dispensed from public pharmacies in Belgium. Pharmanet data include information on the date of dispensing, the quantity per package, the daily defined dose and the national code number of the medicine which allows to link each medicine to its ATC-code.

**Fig. 1:**
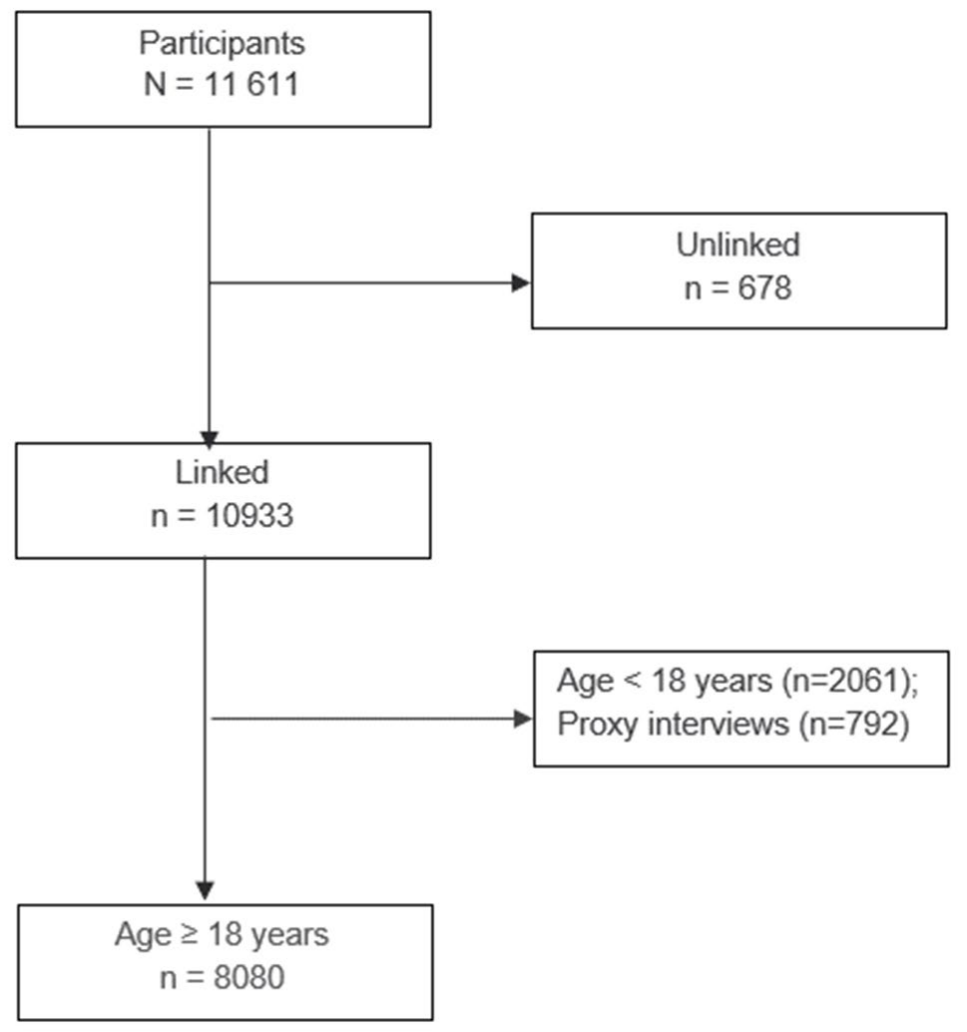
Participants’ selection process for mediation analysis, HISlink 2018, Belgium

Of the total of 11,611 individuals who participated in the BHIS 2018, the linkage was possible for 10,933, resulting in an overall linkage rate of 94%. In the BHIS, questions on HL were only addressed to people aged 15 years and over, in the form of self-report. Because younger individuals may be dependent of their parents’ lifestyle and literacy in health and because the HL instrument was validated for people aged 18 years and over, this study is limited to adults aged 18 years or more (*n* = 8080), except for breast cancer screening (recommended for women aged 50–69 years, *n* = 1261) and flu vaccination (recommended for the 65 years or older, *n* = 1540). Proxy interviews (i.e., a person belonging or not to the household is allowed to respond on behalf of the selected participant, because this participant - for a certain reason – is unable to reply her/him-self) were excluded, Fig. 1.

## Measures

### Dependent variables – health-related outcomes

Health-related outcomes included in this study were either retrieved from the BCHI data (preventive dental care use, breast cancer screening, vaccination against flu among older people, purchase of antibiotics and antidepressants) or from the BHIS data (perceived health status, physical activity, diet, alcohol and tobacco consumption). The purchase of antidepressants was used as a proxy for depression (mental health status). A detailed variable description and operationalization is found in Table 1.

**Table 1:** Variables description and operationalization, HISlink 2018, Belgium

Variables name	Variable description / operationalisation	Data source and timeframe
Dependent variables – Health related outcomes		
*Preventive dental care among adult population aged 18 years and over*	The selected indicator is the proportion of the adult population aged 18 years and over who had at least one contact with a dentist in the reference period, i.e. in 2018, for preventive care such as an oral examination, a prophylactic cleaning, scaling, etc. The specific NIHDI nomenclature codes for the preventive dental care can be found in [32].	BCHI, 2018
*Purchase of antibiotic among population aged 18 years and over*	This indicator is defined as the proportion of the population aged 18 years and over with at least one purchase of antibiotics between 01/07/2018 and 30/06/2019. Pharmanet data were used to identify cases of purchase of antibiotics. Purchase of a prescribed antibiotic was defined as having obtained at least one reimbursement of prescribed medicine belonging to ATC-code group J01 (antibacterials for systemic use) purchased from a public pharmacy (see Table A1 in the supplementary file). As antibiotic purchase has probably a seasonal pattern, there may be more than one peak in antibiotics use in a calendar year. Therefore in order to include only one winter peak per 12-month period, instead of the months January to December, we used the period from July 01, 2018 to June 30, 2019 to express the annual antibiotic purchase [33].	BCHI, 2018–2019
*Vaccination against flu among community dwelling older people aged 65 years and older*	The indicator expresses the proportion of the population aged 65 years and over that is vaccinated against flu in the reference period, i.e., calendar year 2018. Older people aged 65 years and over residing in an institution (rest homes and the rest and care homes) were excluded because in the BCHI data only vaccines which have been reimbursed are taken into account and since 2010 vaccines are free of charge for older people residing in an institution in Flanders [34]. Hence the calculations for this indicator may result in an underestimation of the true coverage rate. All vaccines belonging to the ATC 4 class J07BB (anti-influenza vaccines) were considered.	BCHI, 2018
*Mental health*	The purchase of antidepressants is used as a proxy of mental health. The indicator expresses the percentage of adults aged 18 years and over with at least one purchase of an antidepressant [34] (ATC code = N06A) in 2018.	BCHI, 2018
*Breast cancer screening among women aged 50–69 year in 2018*	Proportion of women aged 50–69 having received at least one mammogram within the last two years, i.e., within the reference year or the reference year-1. In the BCHI data source, the mammographies realized within the screening programme follow a specific procedure, and have their own billing codes. However, these codes do not allow to sufficiently discriminate screening within the program from the other mammographies (opportunistic screening, diagnostic evaluation). Therefore, in this study, all mammograms are considered, within or outside the context of the organised screening programme and we assumed that the largest part of the mammographies undergone between 50 and 69 is made for screening purposes, and therefore we used this information as a proxy of the breast cancer screening. The NIHDI nomenclature codes used can be found in Table A1 in the supplementary file.	BCHI, 2017–2018
*Perceived health status among population aged 18 years and over*	Perceived health status is based on the single question: “How is your health in general?”. This question is part of the Minimum European Health Module (MEHM), which is internationally used. Five response categories are possible: Very good / Good / Fair / Poor / Very poor. The response categories Very good / Good are recorded as “Good” and those Fair / Poor / Very poor as “Poor”.	BHIS, 2018
*Physical activity among population aged 18 years and over*	This refers to non-work-related physical activity (leisure-time physical activity and/or the use of a bicycle for commuting) meeting WHO recommendations: spend at least 150 min per week in physical activities of at least moderate intensity. The Physical Activity Questionnaire developed by European Health Interview Survey (EHIS-PAQ) was used to assess physical activity. This is a dichotomous variable (Practice of physical activity / No practice of physical activity).	BHIS, 2018
*Type of diet among population aged 18 years and over*	The type of diet was assessed using a short food frequency questionnaire. The indicator refers to the proportion of the population aged 18 years and over who eat the recommended daily amount of fruit and vegetables, i.e., at least 5 portions fruits and vegetables (Healthy diet) or not (Unhealthy diet).	BHIS, 2018
*Consumption of alcohol among population aged 18 years and over*	The EHIS wave 3 questions [35] are used to measure alcohol consumption in order to comply to the European Regulation which recommends the use of a harmonized approach in all EU Member States. The indicator expresses the drinking frequency in the past 12 months preceding the survey: Daily / Weekly / Monthly / Less than monthly / None. These categories are dichotomized as: at least once a week/less than once a week) among the population aged 18 years.	BHIS, 2018
*Consumption of tobacco among population aged 18 years and over*	*Proportion of the population aged 18 and over who currently smoke (daily or occasionally).* The tobacco consumption is a dichotomous variable (Yes / No).	BHIS, 2018
**Independent variables**		
Educational attainment	Educational attainment is based on the highest level of education achieved in the household. Possible values are “primary or no degree”, “secondary inferior”, “secondary superior”, and “superior education” following the ISCED-11 classification, whereby superior education includes all obtained degrees higher than secondary superior [36]. These values are recorded into two categories for the analyses: higher secondary education or lower (“primary or no degree”, “secondary inferior”, “secondary superior”) and higher education (“superior education”).	BHIS, 2018
Household income level	The quintiles of the equivalent household income (quintile 1: <750, quintile 2: 751–1000, quintile 3: 1001–1500, quintile 4: 1501–2500, quintile 5: >2500) were recoded in low (quintile 1–3) and high (quintile 4 and 5).	BHIS, 2018
**Mediator variable**		
Health literacy (HL) *among population aged 18 years and over*	The HL level was assessed in the BHIS 2018, using the 6-items European Health Literacy Survey Questionnaire (HLS-EU-Q6), which is a short- form of the original 47-items tool (HLS-EU-Q47) [31]. Like the original, the HLS-EU-Q6 is a self-reported tool whereby participants are asked how easy or difficult they find it to perform an information-related task, using Likert-type responses (“very easy” = 4; “fairly easy” = 3; “fairly difficult” = 2; “very difficult” = 1. “Don’t know” or refusal were recoded as missing. The six items covered are: • Judge when you may need to get a second opinion from another doctor • Use information the doctor gives you to make decisions about an illness • Find information on how to manage certain mental health problems like stress or depression • Judge if the information on health risks in the media is reliable? (Examples: TV, Internet or other media) • Find out about activities that are good for your mental well-being? (Examples: meditation, sport, walking,…) • Understand information in the media on how to get healthier? (Examples: Internet, newspapers, magazines). The scale final score measuring HL is the mean value on the six items, which varies between 1 and 4. Only respondents who answered at least 5 items were considered. Based on the final score, three possible levels of HL are defined: insufficient level of HL (1 ≤ x ≤ 2); limited level of HL (2 < x < 3); sufficient level of HL (3 ≤ x ≤ 4). In this study, HL was a dichotomous variable grouping together insufficient and limited levels of HL as “low HL” - vs. ”sufficient level of HL”.	BHIS, 2018
**Confounding variables**		
Age	Respondents age (in years)	BHIS, 2018
Sex	Respondents gender (Male / Female)	BHIS, 2018

### Independent variables – socioeconomic status

SES is commonly captured by three proxy measures: education, occupation and/or income, or may be constructed as a composite measure of those variables [28, 29]. In this study, educational attainment and income extracted from the BHIS were utilized as proxy indicators for SES. These variables have frequently been used as indicators of SES in previous studies [8, 9, 14, 15, 30]. Other indicators such as occupation [9, 30] and race/ethnicity [8, 15] were not considered here because of data quality or lack of information.

### Mediator variable

The HL level of the Belgian population was assessed via the Belgian BHIS in 2018, using the 6-items European Health Literacy Survey Questionnaire (HLS-EU-Q6), a short-short form of the original 47-items HL questionnaire (HLS-EU-Q47) [31]. Like the original, the HLS-EU-Q6 is a self-reported tool for which participants are asked to indicate how easy or difficult they find it to perform an information-related task (e.g., “judge when you may need to get a second opinion from another doctor”, “use information the doctor gives you to make decisions about an illness”), using Likert-type responses. Detailed information on the construction of the HL level is found in Table 1. Based on the final score, three possible levels of HL are defined: insufficient, limited and sufficient level of HL. In this study, HL was treated as a dichotomous variable grouping together insufficient and limited as insufficient HL vs. sufficient HL.

### Confounding variables

Based on previous studies, the demographic characteristics that were identified as potential confounders in the assessment of the association between SES and health outcomes were sex (male/female) and age (in years as a continuous variable) [9, 10, 14, 30].

## Statistical analysis

### Descriptive analysis

Descriptive statistics summarizing the sociodemographic characteristics of the participants are presented as a percentage in case of categorical variables and as a mean in case of continuous variables. Participants’ characteristics were estimated overall and by level of HL. Comparisons were statistically tested using a χ2 test for categorical variables and a t-test for normally distributed continuous age variable. In addition, the association between SES and health related outcomes was tested after controlling for age and sex in a regression analysis. Only associations that remained significant after adjusting for age and sex were considered for the mediation analysis.

### Mediation analysis

To test the hypothesis that HL is a pathway through which educational attainment and household income affect the selected health related outcomes, the mediation effect of HL was examined separately for each of the two SES factors considered [9] and for each of the selected outcomes.

**Fig. 2:**
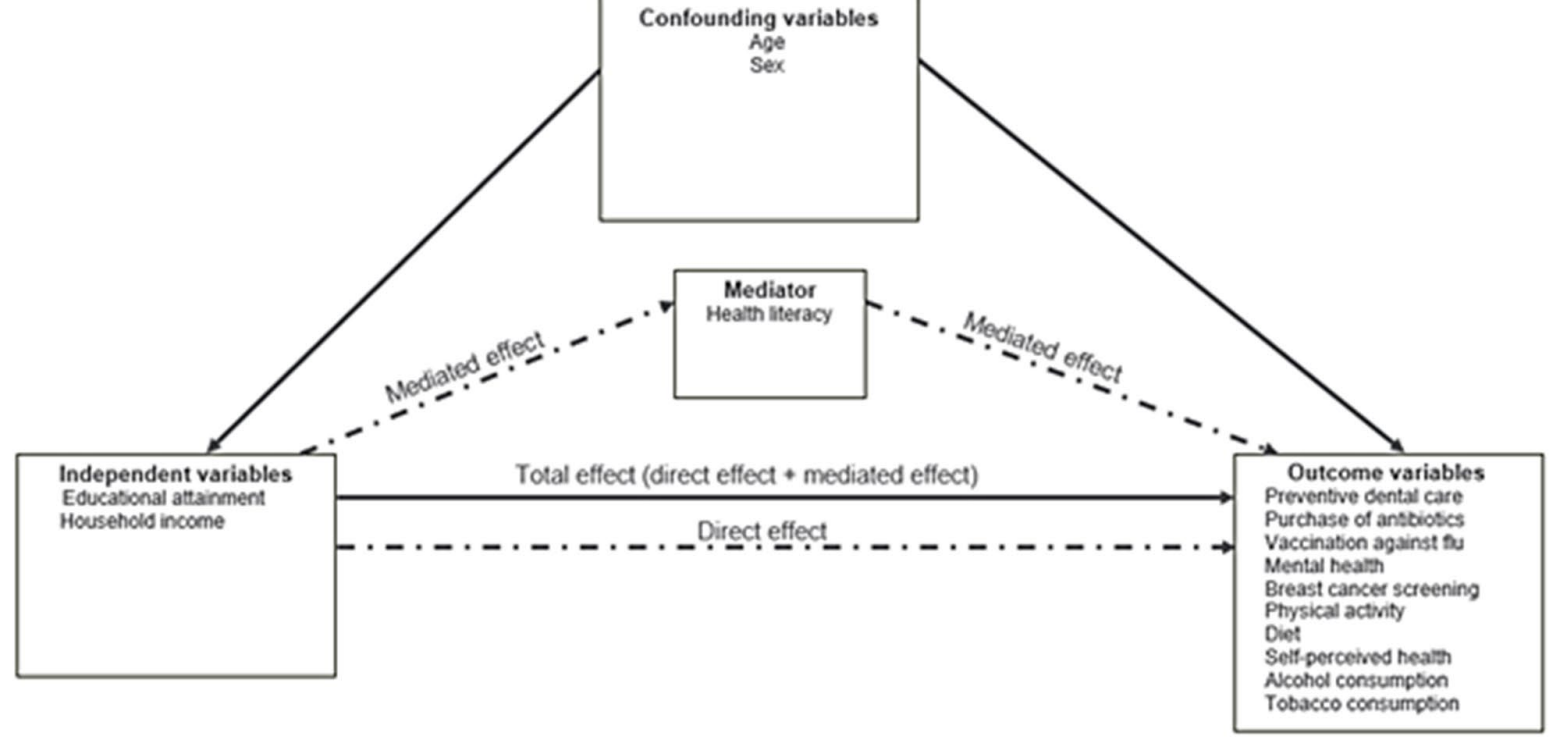
Conceptual model of HL as a mediator of the association between SES factors and health related outcomes, HISlink 2018, Belgium

The analysis proceeded in two steps. First, two logistic regression models were specified: [1] the mediator model for the conditional distribution of the mediator (HL) given the independent variable (SES), and [2] the outcome model for the conditional distribution of the outcomes given the independent variable and the mediator. These models were fitted separately and controlled for age and sex as covariates (except for breast cancer screening where the model was controlled for age only) because they were expected to be all related to the key variables (see Fig. 2 for the conceptual model). Age was entered as a continuous variable, whereas sex, HL and SES were dichotomous variables [9]. The outcome model also contained an interaction term for the independent variables x the mediator [9, 32]. By including an interaction term, we assume that the odds ratio (OR) comparing categories of SES differs according to the mediator variable, i.e., HL, and vice versa. The outputs from the mediator and outcome regression models served as the main inputs to estimate the causal effects for the single mediator model [9, 32–34]. Missing values (proportion ranged from 0.12 to 14.5%) were imputed using the fully conditional specification method. As all the variables with missing values are either binary or ordinary, a logistic regression method was used to impute missing values [35]. Age, sex, region of residence, health-related outcome variables as well as survey weights and strata were used in the imputation model. We created 20 imputed data sets. This number was large enough to achieve a very good efficiency.

A sensitivity analysis was carried out for the purchase of antidepressants (using a threshold of 90 DDD per year of specific medication ATC codes to take into account the quantity of antidepressants purchased).

All analyses were performed using SAS® (version 9.4), taking into account the survey weights for the descriptive analysis. The Causalmed procedure was used for the mediation analysis [33, 36]. Bootstrap methods (1000 bootstrapped samples) were used to compute standard errors and confidence intervals for causal mediation effects and decompositions [10, 13, 36, 37]. The Causalmed procedure computes the total effect of the independent variable on the outcome and decomposes this effect into the indirect and direct effects [36]. In terms of interpretation, the indirect effect reflects the magnitude of the effect that is transmitted through the mediator, whereas the direct effect accounts for all the other possible causal chains. Furthermore, the Causalmed procedure yields the proportion mediated, which should be interpreted as an estimate of the percentage of the total effect that is exerted through the mediator [14, 30, 33, 36] and provide insight into the relative importance of the mediating role of HL. For each analysis, an α level below 0.05 was considered as significant. All P values are two-tailed.

This study is reported according to the STROBE cross sectional reporting guidelines [38].

## Results

### Descriptive statistics

#### Participants characteristics

Participants characteristics are presented in Table 2. The crude *n*, unweighted percentages are presented as well as weighted percentages to match the distribution of the population in terms of age, sex and region of residence. Females represented 52.0% of the adult population and the mean age is 50.5 years old (SD = 0.3). Less than one participants out of two was higher educated (46.8%). As for income, 52.2% of the participants belonged to a household with higher income category. In terms of HL, sufficient level of HL was found in 65.8% of the population. People who had a sufficient level of HL were more likely to be male, higher educated, and belong to a high income household. Further characteristics are found in Table 2.

**Table 2:** Participants characteristics overall and by level of health literacy, *n* = 8080, HISlink 2018, Belgium

	Total			Sufficient level of HL			Insufficient level of HL			*P* value
	*N*	Unweighted % (sample)	Weighted % (population)	*n*	Unweighted % (sample)	Weighted % (population)	*n*	Unweighted % (sample)	Weighted % (population)	
**All**	8080	100	100	5255	66.0	65.8	2825	35.0	34.2	
**Sex**										*0.0141*
Male	3812	47.2	48.0	2517	66.0	67.2	1295	34.0	32.8	
Female	4268	52.8	52.0	2738	64.1	64.5	1530	35.9	35.5	
**Age, mean ± SE**	8080	51.8 ± 18.0	50.5 ± 0.3	5255	51.8 ± 17.5	50.5 ± 0.4	2825	52.0 ± 19.0	50.5 ± 0.6	*0.9414*
**Educational attainment**										
Higher secondary education or lower	4243	52.5	53.2	2527	59.6	60.3	1716	40.0	39.7	< 0.0001
Higher education	3837	47.5	46.8	2728	71.1	72.1	1109	28.9	27.9	
**Income**										< 0.0001
Lower income	4149	51.3	47.8	2532	61.0	61.1	1617	39.0	38.9	
Higher income	3931	48.7	52.2	2723	69.3	70.1	1208	30.7	29.9	

### Prevalence of health outcomes

FigureA1 in the supplementary file illustrates the prevalence of health outcomes overall and by HL level. Overall, the rates prevalences range from 13.0% for the purchase of antidepressants to 76.7% for perceived good health, and vary most often by HL level.

### Association between health literacy, educational attainment, household income and health related outcomes

#### Association between HL and SES

Lower educational attainment and to a lesser extent lower income are associated with having an insufficient level of HL (Table 3).

**Table 3:** Association between HL (*Insufficient level of HL” vs. “Sufficient level of HL*) and independent variables, HISlink 2018, Belgium

	Odds Ratio^a^ (95% CI)		
	Subgroup aged 18 years and over^b^	Women aged 50–69 years^c^	Subgroup aged 65 years and over^d^
**Educational attainment**			
Higher secondary education or lower	1.69 (1.53–1.86)***	2.09 (1.63–2.67)***	1.98 (1.57–2.50)***
Higher education	1	1	1
**Income category**			
Lower income	1.45 (1.30–1.64)***	1.55 (1.17–2.04)**	1.71 (1.33-2.00)***
Higher income	1	1	1

^a^ Adjusted by age and sex, for breast cancer screening, the OR is adjusted for age only; ^b^ for physical activity, diet, alcohol consumption, tobacco consumption

Perceived health, mental health, purchase of antibiotics and preventive dental care; ^c^ for breast cancer screening; ^d^ for vaccination against flu; ** *p* < 0.05; *** *p* < 0.0001

### Association between SES and health related outcomes

#### Association between SES and health behaviour

Lower educational attainment and lower income are associated with lower likelihood of being physically active, having a healthy diet, and reporting weekly alcohol consumption. In contrast, lower educational attainment and lower household income are associated with a higher likelihood of reporting tobacco consumption (Tables 4 and 5 for educational attainment and income respectively).

**Table 4:** Association between health literacy, educational attainment and health related outcomes, HISlink 2018, Belgium

	Odds Ratio^a^ (95% CI)									
	Health behaviour				Health status		Use of medicine	Preventive health care		
	Physical activity	Healthy diet	Alcohol consumption (At least once a week)	Tobacco consumption (Current smokers)	Good perceived health	Poor mental health	Purchase of antibiotics	Preventive dental care	Vaccination against flu	Breast cancer screening
**Health literacy**										
Insufficient level of HL	0.79 (0.68–0.93)**	0.72 (0.58–0.89)**	0.86 (0.74–0.99)**	1.25 (1.03–1.53)**	0.56 (0.46–0.68)***	1.51 (1.21–1.88)**	1.03 (0.88–1.20)	0.99 (0.85–1.15)	1.25 (0.86–1.81)	0.89 (0.60–1.33)
Sufficient level of HL	1	1	1	1	1	1	1	1	1	1
**Educational attainment**										
Higher secondary education or lower	0.54 (0.48–0.62)***	0.49 (0.41–0.58)***	0.40 (0.36–0.45)***	1.85 (1.59–2.15)***	0.51 (0.44–0.59)***	1.37 (1.15–1.63)**	1.20 (1.01–1.29)**	0.48 (0.43–0.55)***	1.07 (0.84–1.35)	0.64 (0.49–0.84)**
Higher education	1	1	1	1	1	1	1	1	1	1
Educational attainment and HL interaction term	0.89 (0.70–1.13)	0.93 (0.67–1.29)	1.00 (0.82–1.23)	0.96 (0.75–1.23)	0.89 (0.70–1.13)	0.98 (0.73–1.31)	1.09 (0.87–1.36)	0.70 (0.51–0.95)**	0.89 (0.57–1.40)	1.00 (0.60–1.66)

^a^ Adjusted by age and sex, for breast cancer screening, the OR is adjusted for age only; ** *p* < 0.05; *** *p* < 0.0001

**Table 5:** Association between health literacy, income and health related outcomes, HISlink 2018, Belgium

	Odds Ratio^a^ (95% CI)									
	Health behaviour				Health status		Use of medicine	Preventive health care		
	Physical activity	Healthy diet	Alcohol consumption (At least once a week)	Tobacco consumption (Current smokers)	Good perceived health	Poor mental health	Purchase of antibiotics	Preventive dental care	Vaccination against flu	Breast cancer screening
**Health literacy**										
Insufficient level of HL	0.76 (0.65–0.89)**	0.68 (0.55–0.85)**	0.80 (0.69–0.93)**	1.22 (1.02–1.47)**	0.53 (0.43–0.65)***	1.45 (1.14–1.85)**	1.08 (0.91–1.28)	0.96 (0.83–1.12)	1.60 (1.04–2.45)**	0.90 (0.60–1.35)
Sufficient level of HL	1	1	1	1	1	1	1	1	1	1
**Income category**										
Lower income	0.69 (0.60–0.78)***	0.78 (0.67–0.92)**	0.50 (0.44–0.57)***	1.64 (1.39–1.92)***	0.50 (0.42–0.58)***	1.45 (1.19–1.78)**	1.06 (0.93–1.20)	0.59 (0.52–0.68)***	1.02 (0.78–1.34)	0.54 (0.41–0.71)***
Higher income	1	1	1	1	1	1	1	1	1	1
Income and HL interaction term	0.89 (0.71–1.13)	0.90 (0.65–1.25)	1.03 (0.84–1.27)	1.05 (0.81–1.35)	0.93 (0.72–1.20)	1.06 (0.77–1.45)	1.03 (0.83–1.28)	0.85 (0.69–0.99)**	0.66 (0.39–1.11)	0.97 (0.58–1.59)

^a^ Adjusted by age and sex, for breast cancer screening, the OR is adjusted for age only; ** *p* < 0.05; *** *p* < 0.0001

#### Association between SES and health status

Lower educational attainment and lower income are associated with a lower likelihood of reporting good perceived health status. Lower educational attainment and lower income are related to a higher likelihood of having a poor mental health (Tables 4 and 5 for educational attainment and income respectively).

#### Association between SES and use of medicine

Lower educational attainment is associated with higher likelihood of purchase of antibiotics. No significant association is observed between income and the purchase of antibiotics (Tables 4 and 5 for educational attainment and income respectively).

#### Association between SES and use of preventive care

Lower educational attainment and lower income are associated with lower likelihood of receiving preventive dental care and breast cancer screening. No significant association is observed between both SES and vaccination against flu (Tables 4 and 5 for educational attainment and income respectively).

### Association between HL and health related outcomes

HL is positively associated with physical activity, diet and alcohol consumption. In contrast, HL is negatively associated with tobacco consumption. Insufficient level of HL is associated with poor perceived health status and poor mental health status. An insufficient level of HL in the low SES group is associated with a lower likelihood of preventive dental care use. In contrast, insufficient level of HL is associated with a greater likelihood of vaccination against flu. No significant association is observed between HL and purchase of antibiotics and participation in breast cancer screening (Tables 4 and 5 for educational attainment and income model respectively).

### Mediation effect of health literacy

#### Mediation effect of HL on the relationship between educational attainment and health related outcomes

Table 6 presents the results of mediation analysis (results from multiple imputation).

**Table 6:** Mediation effects of health literacy (reference = sufficient level of health literacy) in the relationship between health related outcomes^a^ and educational attainment (reference = higher education), HISlink 2018, Belgium – Results from multiple imputation

	Odds Ratio^b^ (95% CI)
**Health behaviour**	
**Practice of physical activity vs. No practice of physical activity**	
Total Effect	0.51 (0.45–0.56)***
Direct effect	0.53 (0.47–0.59)***
Indirect effect	0.96 (0.94–0.98)**
Percentage mediated (%)	4.1 (1.7 to 6.5)**
**Healthy diet vs. Unhealthy diet**	
Total Effect	0.46 (0.40–0.52)***
Direct effect	0.48 (0.41–0.56)***
Indirect effect	0.96 (0.93–0.98)**
Percentage mediated (%)	3.8 (1.2 to 6.5)**
**Alcohol consumption (At least once a week vs. Less than once a week)**	
Total Effect	0.39 (0.36–0.43)***
Direct effect	0.40 (0.36–0.44)***
Indirect effect	0.98 (0.96–0.99)**
Percentage mediated (%)	1.1 (0.1 to 2.2)
**Tobacco consumption (current smokers vs. No current smokers)**	
Total Effect	1.87 (1.64–2.09)***
Direct effect	1.83 (1.60–2.05)***
Indirect effect	1.02 (1.01–1.04)**
Percentage mediated (%)	4.8 (0.4 to 9.3)
**Health status**	
**Good perceived health vs. Poor perceived health**	
Total Effect	0.46 (0.41–0.52)***
Direct effect	0.50 (0.43–0.56)***
Indirect effect	0.93 (0.91–0.95)***
Percentage mediated (%)	6.4 (3.9 to 9.0)***
**Poor mental health status**	
Total Effect	1.43 (1.24–1.61)***
Direct effect	1.36 (1.18–1.54)**
Indirect effect	1.05 (1.02–1.08)**
Percentage mediated (%)	16.0 (5.2 to 26.7)**
**Preventive health care**	
**Preventive dental visit vs. No preventive dental visit**	
Total Effect	0.46 (0.42–0.51)***
Direct effect	0.47 (0.42–0.51)***
Indirect effect	0.98 (0.97-1.00)
Percentage mediated (%)	1.3 (-0.3 to 2.9)

^a^ Certain health related outcomes were not included because after controlling for confounding factors, the association between these outcomes and education or health literacy was no longer significant; ^b^ Adjusted by age and sex; Bootstrap Percentile 95% Confidence Limits; ** *p* < 0.05; *** *p* < 0.0001. All P values are two-tailed

#### Health behaviour

On average, HL is found to significantly mediate the associations between educational attainment and all the health behaviours considered, i.e., physical activity (OR of indirect effect = 0.96, 95% CI: 0.94–0.98), diet (OR of indirect effect = 0.96, 95% CI: 0.93–0.98), alcohol consumption (OR of indirect effect = 0.98, 95% CI: 0.96–0.99) and tobacco consumption (OR of indirect effect = 1.02, 95% CI: 1.01–1.04). The percentage mediated was 4.1% and 3.8% for physical activity and diet, respectively and is not significant for alcohol and tobacco consumption.

#### Health status

HL mediates the association between educational attainment and perceived health status (OR of indirect effect = 0.93, 95% CI: 0.91–0.95), mental health (OR of indirect effect = 1.05, 95% CI: 1.02–1.08) accounting for 6.4% and 16.0% of the total effect, respectively.

#### Preventive health care

No significant mediating role of HL is found for the relationship between educational attainment and preventive dental care (OR of indirect effect = 0.98, 95% CI: 0.97-1.00).

Overall, the results from multiple imputation are in line with those from complete case analysis (Table A2 in supplementary file).

### Mediation effect of HL in the relationship between income and health related outcomes

Table 7 presents the results of mediation analysis (from multiple imputation).

**Table 7:** Mediation effects of health literacy (reference = sufficient level of health literacy) in the relationship between health related outcomes^a^ and household income (reference = higher household income), HISlink 2018, Belgium – Results from multiple imputation

	Odds Ratio^b^ (95% CI)
**Health behaviour**	
**Practice of physical activity vs. No practice of physical activity**	
Total Effect	0.65 (0.58–0.72)***
Direct effect	0.67 (0.59–0.74)***
Indirect effect	0.97 (0.95–0.98)**
Percentage mediated (%)	5.8 (2.0 to 9.6)**
**Healthy diet vs. Unhealthy diet**	
Total Effect	0.74 (0.63–0.85)***
Direct effect	0.77 (0.65–0.88)***
Indirect effect	0.96 (0.94–0.98)**
Percentage mediated (%)	10.9 (4.0 to 25.8)**
**Alcohol consumption (At least once a week vs. Less than once a week)**	
Total Effect	0.50 (0.45–0.55)***
Direct effect	0.51 (0.45–0.56)***
Indirect effect	0.98 (0.97–0.99)**
Percentage mediated (%)	1.5 (0.2 to 2.8)
**Tobacco consumption (current smokers vs. No current smokers)**	
Total Effect	1.70 (1.48–1.92)***
Direct effect	1.66 (1.44–1.88)***
Indirect effect	1.02 (1.01–1.04)**
Percentage mediated (%)	5.2 (1.3 to 9.1)**
**Health status**	
**Good perceived health vs. Poor perceived health**	
Total Effect	0.46 (0.40–0.52)***
Direct effect	0.49 (0.42–0.53)***
Indirect effect	0.95 (0.93–0.97)***
Percentage mediated (%)	4.7 (2.5 to 6.9)**
**Poor mental health status**	
Total Effect	1.54 (1.30–1.78)***
Direct effect	1.48 (1.25–1.71)***
Indirect effect	1.04 (1.02–1.06)**
Percentage mediated (%)	10.8 (3.7 to 17.9)**
**Preventive health care**	
**Preventive dental visit vs. No preventive dental visit**	
Total Effect	0.56 (0.50–0.62)***
Direct effect	0.57 (0.51–0.63)***
Indirect effect	0.98 (0.97–0.99)**
Percentage mediated (%)	2.1 (0.7 to 4.0)**

^a^ Certain health related outcomes were not included because after controlling for confounding factors, the association between these outcomes and income or health literacy was no longer significant ; ^b^ Adjusted by age and gender, Bootstrap Percentile 95% Confidence Limits; ** *p* < 0.05; *** *p* < 0.0001. All P values are two-tailed

#### Health behaviour

HL significantly mediates the association between income and physical activity (OR of indirect effect = 0.97, 95% CI: 0.95–0.98), diet (OR of indirect effect = 0.96, 95% CI: 0.94–0.98), alcohol consumption (OR of indirect effect = 0.98, 95% CI: 0.97–0.99) and tobacco consumption (OR of indirect effect = 1.02, 95% CI: 1.01–1.04). The percentage mediated ranges from 5.2 to 10.9% and was not significant for alcohol consumption.

#### Health status

A mediating role of HL is found for the association between income and perceived health status (OR of indirect effect = 0.95, 95% CI: 0.93–0.97). The indirect effect accounts for 4.7% of the total effect. HL significantly mediates the association between income and mental health status (OR of indirect effect = 1.04, 95% CI: 1.02–1.06), accounting for 10.8% of the total effect of income. In sensitivity analysis, even taking into account a threshold of 90 DDD of antidepressants, the mediating effect of HL in the relationship between income and mental health status remains significant (OR of indirect effect = 1.04, 95% CI: 1.02–1.07). The percentage mediated is about 12.7% (see Table A4 in the supplementary file).

#### Preventive health care use

HL acts as mediator in the relationship between income and use of preventive dental care, (OR of indirect effect = 0.98, 95% CI: 0.97–0.99), accounting for 2.1% of the variance.

Overall, the results from multiple imputation are in line with those from complete case analysis (Table A3 in supplementary file).

## Discussion

### Main findings

The reduction of SE health disparities is an important objective for public health policies. It is therefore relevant to identify factors that contribute to these disparities. There is a growing body of research to suggest that HL may be an explanatory factor in pathways that generate health disparities, especially those associated with social determinants of health. This study explored whether HL acts as a mediator in the association between SES as measured by educational attainment and household income and the selected health related outcomes that are of interest from a public health perspective.

The SE disparities in health outcomes are confirmed with our data. HL was found to partly mediate the association between educational attainment and health behaviour, and between educational attainment perceived health status, and mental health. HL constitutes a pathway through which income influences health behaviour, perceived health status, mental health status and preventive dental care.

As expected, a mediation effect of HL for the link with SES was found in all of the health behaviours considered. Although the contributing effect of HL to the total effect is rather small, it is in line with the existing evidence [13, 22]. Indeed, in a Danish population-based study, Friis et al. (2016) found that HL mediated the relationship between educational attainment and health behavior, especially in relation to being physically inactive (accounting for 5.4–20% of the variance depending of the scales from HL questionnaires), having a poor diet (accounting for 13% of the variance), and daily smoking (accounting for 4.5–6.6%) [22]. Although using different independent variable, Chen et al. (2019) demonstrated that HL played a partial mediating role between social capital and physical activity (8.2–12.7% of the total effect) as well as type of diet (4.93–12.7% of the total effect) [13].

Compared with the other health behaviours studied, the mediating role of HL in the relationship between SES and alcohol and tobacco consumption is relatively limited. Indeed, although a significant mediating effect can be found, the contribution of HL in terms of the percentage mediated is not significant (except between tobacco consumption and income). In previous study, Friis et al. (2016) did not find a mediation effect of HL in the association between education and tobacco consumption. The authors argued that the underlying explanations for this may be link to the fact that in Denmark policy regulations and mass media campaigns relating to tobacco use have been in place for more than two decades. So, regardless of their HL levels, most people are aware of the health-related consequences of smoking [22]. A similar result was found by Van Den Broucke et al. (2014) [39]. The underlying hypothesis put forward by Friis et al. (2016) could be applied to our findings, because an anti-smoking plan introduced legislative measures in Belgium since 2006 that include, for example, increase in tobacco price, banning smoking in public place and dissuasive colour photos. These measures are likely to have an impact on the risk of individuals’ tobacco consumption, whatever their level of HL [40].

The mediation effect of HL was found for the association between educational attainment and perceived health status, suggesting that low educated people manage their health problems less well, resulting in poorer perceived health status. Therefore, a better HL among low educated people will lead to a better perceived health status for them. This result is in line with results from previous studies [9]. Some studies have shown that the relative importance of HL as a pathway between education and perceived health status is greater among people with lower levels of education than among those with higher levels of education [9, 14], but Van Heide et al. (2013) also found that the mediating role of HL does not show a linear gradient as education level increases [14]. This means that HL exhibited a more important pathway for lower secondary educated than for preprimary/primary educated [14]. In the present study, we were unable to explore this issue as we only used two levels of education. To determine the extent to which improving HL could help reduce education-related disparities in health status, further research is needed on the relative importance of the mediating role of health literacy between different levels of education.

With regards to mental health status, we found that the association with both SES is mediated by HL. These results could be explained by the fact that, unlike people with a sufficient level of HL, people with an insufficient level of HL do not know or understand that they can consult a psychologist for their mental health problems and therefore turn to the use of antidepressants. Furthermore, in Belgium, it is less expensive to take antidepressants (which are fully reimbursed) than to undergo therapy (which is not reimbursed).

Finally, with regard to preventive health care, HL significantly mediated the association between income and preventive dental care. The vaccination against flu and participation in breast cancer screening were not considered for mediation analysis because after controlling for participants’ age and sex, the association between these indicators, SES and/or HL was no longer significant. These findings may be linked to the universal health care system that is in place in Belgium. As suggested by previous studies [30, 41], in countries with universal, publicly-funded health care systems, the burden exerted by SES or HL is small or absent, since it is reduced by an equitable access, free of charge, for all the target categories of the population. Therefore, individual decisions are not likely to play a crucial role in this behaviour, and so the influence of HL may be minimal.

### Strengths and limitations

To our knowledge, this is the first study based on the linkage of two population databases to examine whether HL plays a mediating role in the associations between education, income and a number of objective and subjective measures of health related outcomes in different domains, namely health behaviour, health status, and use of medicine and preventive care in a large sample. Studies most often rely on subjective measures to this respect. However, it has been recognised that to better understand the association between HL and health outcomes, objective measures of the latter may provide important evidence [14] and should therefore be used wherever possible. The use of causal mediation and the inclusion of the interaction between the mediator and SES in the model are another strength of the current study. In addition, multiple imputation has enabled us to deal carefully with missing values, thus avoiding the bias associated with them and maintaining the statistical power of our analytical sample.

Our study has a number of limitations that must be acknowledged. First, using the criterion of purchasing at least one prescription of antidepressants in the reference period to identify cases of mental health may have caused the inclusion of individuals who use antidepressants for another indication than depression, who did not comply with or respond to the treatment. However, the results from the sensitivity analysis taking into account a threshold of 90 DDD per year of specific medication ATC codes confirmed the mediation effect of HL, meaning that our indicator was accurate. On the other hand, the use of antidepressants can also be a limitation, as mental health goes beyond the simple use of antidepressants. However, the prevalence of mental health status found in our study is consistent with that found by Van Heide et al. (2013) using self-reported mental health status [14].

A second limitation is that regarding breast cancer screening, no distinction was made between mammographies as part of a screening program and opportunistic mammographies. Even though the mammographies realized within the program have their own billing codes in the BCHI data, they do not sufficiently discriminate screening within the program from the other mammographies (opportunistic screening, diagnostic evaluation). In fact, opportunistic screening mammograms are often miscoded as diagnostic mammograms for reimbursement purposes in the BCHI. However, we assumed that the largest part of the mammographies undergone between 50 and 69 years of age is made for screening purposes, giving information as to preventive health care initiatives.

Third, the instrument that was used to assess HL in this study was a generic one, which may explain the relatively low percentage of mediated effects that were found. In fact, some authors suggest the use of outcome-specific health literacy instruments (e.g., vaccine literacy) to better assess the role of for decision making in that field [30]. However, our instrument is validated and has good validity. The next survey BHIS 2023 includes a more extensive HL instrument (12-item questionnaire) [42, 43] and will allow us to verify our findings.

Fourth, the dichotomisation of the HL level and SES may have resulted in a loss of information. For instance, the dichotomisation puts people with different HL levels in one category and “within differences” in each of the categories are not included in the analysis. This may dilute the information of the HL indicator, as a result of which the mediation effect will be underestimated. The results of this study should therefore be interpreted with caution.

Finally, the lack of sensitivity analysis for unmeasured confounders also constitutes a further limitation to this study.

### Implications and future perspective

This study has important implications for practitioners and policy makers. Besides the fact that it adds further insights that help to understand the underlying mechanisms linking SES to health related outcomes, the mediating role of HL may have important implications for interventions that are aimed at reducing health disparities, as HL can be modified via health and literacy programs contrary to SES factors. Policies and interventions aimed at increasing the level of HL in the population or that take people’s insufficient level HL better into account might effectively contribute to reduce health disparities. As this study again demonstrates, the most vulnerable and disadvantaged people in society are more at risk of limited HL and are known to have the poorest health outcomes. Strategies to improve HL are therefore important empowerment tools which have the potential to reduce health disparities.

The role of HL in addressing health disparities has thus far not been at the forefront of HL research. However, while HL has been considered a direct, independent social determinant of health by some [44], a systematic literature review by Stormacq et al. (2019) [15] suggests a partial mediating role for HL in the relationship between social and economic determinants and observed health outcomes, in the sense that HL mediates the association between socioeconomic status and specific health outcomes, health-related behaviors, and access to and use of health services. Since HL is more immediately amenable to change than (structural) social and economic conditions, addressing low HL may be a practical strategy to reduce disparities and promote greater equity in health. That does not necessarily imply placing the responsibility on individuals, because addressing low HL involves making health services more accessible to people with low HL, or even making health organizations more health literate friendly (i.e., promoting organizational HL) as much as strengthening HL in the population. We acknowledge that addressing low HL is often limited to efforts to strengthen HL among patients or in the population, whereas it should also imply making health services and public health systems more accessible to people with low HL by reducing complexity. This need to make the health system more “health literacy friendly”, as a complement to increasing individuals’ health literacy, is particularly emphasized in the Organizational Health Literacy approach, which refers to efforts undertaken by health care organizations to develop and implement strategies to make it easier for patients to understand health information, navigate the health care system, engage in the health care process, and manage their health [45, 46].

Furthermore, while interventions to increase HL or to take people’s low HL into account will not lift people from disadvantaged socioeconomic conditions, it can be considered as a ‘midstream’ strategy to reduce the impact of ‘upstream’ socioeconomic determinants on ‘downstream’ disparities in health [47]. So, while there is some scope to improve health equity through interventions that address low HL, this approach should not be regarded as a substitute for the need to tackle the root causes of inequity and the need to address underlying inequities in the distribution of power, resources, and opportunity.

Several strategies have been proposed for effective improvement of HL such as developing initiatives to increase the level of HL in the population through interventions at several levels (political, institutional, professional, citizen) [48, 49]. For example health literacy interventions in the delivery of Medicine in US in which pharmacists, as healthcare professionals who will dispense prescriptions for medication, have a key role to advise the patient on any queries relating to their medication and to counsel on appropriate use. The mental health literacy interventions in adults in which it is assumed that changing mental health literacy will lead to a change in behaviours that benefit mental health, which will, in turn, produce an improvement in mental health [50]. Another strategy consists of improving the detection of people with a low level of HL and adapting communication during contact with healthcare professionals. To identify people with low health literacy, use can be made of validated tools such as the Brief Health Literacy Screening (BHLS), a short (three items) self-report instrument to identify patients with inadequate HL in clinical settings [51]. Alternatively, some behaviours on the part of patients may suggest low HL, such as : frequently missing appointments (the patient may be unable to read the appointment slip or may not have an organizational system to remember appointment), incomplete registration forms (which may be too complicated for that individual patient), noncompliance with medication therapy (due to a lack of understanding of the importance of the medication), inability to name medications or explain their purpose or dosing, inability to give a coherent sequential history, not asking questions (which may be an indication of not understanding), or lack of follow-through on tests or referrals. Van den Broucke et al. (2018) also highlighted the need to invest in building the capacity of the public health system and of other stakeholders to address health literacy [52]. Empowerment of professionals through training, continuing education and interdisciplinary initiatives to improve health literacy and strengthen communication between the public and professionals has also been identified as an important strategy [53].

In view of the limited contribution of HL in the total effect of SES on the various health related outcomes examined in the current study, and in light with the results of previous studies discussed above [13, 22], it should be noted that in general the influence of HL in the relationship between SES and health related outcomes is rather weak. This may indicate the complexity of health disparities and suggest the influence of other factors or mechanisms that need to be investigated. Indeed, in a new conceptual framework, Schillinger (2021) has described two primary pathways that generate consequences for health outcomes based, in part, on HL. The first pathways is related to the unequal distribution of resources and exposures and their related environmental and public health literacies. The second pathways operates through underdeveloped and discriminatory institutional capacities of the health care systems, and the related individual communicative literacies of the patients that rely on these systems. Both pathways emerge within a complex society characterized by competing forces that reflect both a history of marginalization and oppression of vulnerable subgroups [16]. Furthermore, Paasche-Orlow et al. (2018) also argue that [1] the very society that generates and perpetuates limited literacy is the one that creates a discriminatory healthcare system, and [2] that health and illness (and health disparities) are largely determined by the maldistribution of social and environmental forces and exposures - problems that can be addressed, at least in part, by improving health literacy [54].

Future research should therefore also take other potential mediators into account, such as social support and environmental exposure or other contextual factors. Furthermore, it would be useful to look at mediation effects per stratum (age, sex, cultural background), to allow targeting interventions to specific groups. Zanobini et al. (2022) also suggest to investigate the hypothesis that SES could be the mediator variable between HL and influenza vaccine uptake [30]. Finally, since different HL dimensions show distinct direct and indirect pathways in influencing health outcomes [22, 55], it is necessary to assess the mediating role of HL separately different dimensions. Based on the findings from such investigation, interventions could be targeting dimensions and population subgroups that are at risk. A multiple mediator models could also be considered [56] for identifying these complex underlying mechanisms.

## Conclusion

This study provides evidence that HL partially serves as a pathway thorough which educational attainment and income affect health behaviour, perceived health status, mental health status and preventive dental care. Although the mediating influence of HL in this respect is rather limited, the results suggest that strategies to reduce health disparities in these areas could benefit from taking individuals’ HL into account in awareness campaigns as part of prevention, patient education and other public health interventions. Further data and analysis are needed to confirm our results and to better explore the mediating effects of HL.

### Electronic supplementary material

Below is the link to the electronic supplementary material.

Supplementary Material 1

## Data Availability

The datasets analysed during the current study are not publicly available because they contain sensitive and identifying information, but are available from the corresponding author on reasonable request. Further information regarding the survey and the data access procedure can be found here: Health Interview Survey | Microdata request procedure | sciensano.be.

## Abbreviations

ATC: Anatomical Therapeutic Chemical
BCHI: Belgian compulsory health insurance
BHIS: Belgian health interview survey
CI: Confidence interval
DDD: Daily defined dose
EC: Ethics committee
EHIS: European Health Interview Survey
EHIS-PAQ: Physical Activity Questionnaire developed by European Health Interview Survey
GDPR: General Data Protection Regulation
HIS: Helath Interview Survey
HISLink: Linkage between Belgian Health Interview Survey data and Belgian Compulsory Health Insurance data
HL: Health Literacy
HLS-EU-Q6: European Health Literacy Survey Questionnaire 6-items
IMA: InterMutualistic Agency
ISCED: International Standard Classification of Education
KCE: Belgian Health Care Knowledge Centre
MEHM: Minimum European Health Module
NIHDI: National Institute for Health and Disability Insurance
OR: Odds Ratio
SE: Socioeconomic
SES: Socioeconomic status
WHO: World Health Organization
