# Supplementary material for: Does health literacy mediate the relationship between socioeconomic status and health related outcomes in the Belgian adult population?
Source: BMC Public Health. 2024 Apr 27;24:1182. doi: 10.1186/s12889-024-18676-7 (PMC11055376; doi:10.1186/s12889-024-18676-7)
Supplement: Supplementary file 1 — Supplementary Material 1 [file 12889_2024_18676_MOESM1_ESM.docx]

Table A1 : ATC-codes / Nomenclature codes used for cases definition

| Indicators | ATC-codes / Nomenclature codes |
| --- | --- |
| Purchase of antibiotics | J01A, J01B, J01C, J01D, J01E, J01F, J01G, J01X, J01R, J01X. |
| Vaccination against flu | J07BB |
| Purchase of antidepressants | N06A |
| Mammography | 450096, 450100, 450192, 450203, 461090, 461101. |

Figure A1: Prevalence of health related outcomes overall and by HL level, HISlink 2018, Belgium

Table A2: Mediation effects of health literacy (reference = sufficient level of health literacy) in the relationship between health related outcomes^a^ and educational attainment (reference = higher education), HISlink 2018, Belgium

|  | **Odds Ratio^b^**  **(95% CI)** |
| --- | --- |
| **Health behaviour** |  |
| ***Practice of physical activity vs. No practice of physical activity*** |  |
| Total Effect | 0.58 (0.48-0.60)*** |
| Direct effect | 0.56 (0.51-0.66)*** |
| Indirect effect | 0.96 (0.94-0.98)*** |
| Percentage mediated (%) | 4.6 (2.5 to 7.7)** |
| ***Healthy diet vs. Unhealthy diet*** |  |
| Total Effect | 0.47 (0.40-0.54)*** |
| Direct effect | 0.49 (0.41-0.56)*** |
| Indirect effect | 0.96 (0.93-0.98)** |
| Percentage mediated (%) | 3.7 (1.4 to 6.6)** |
| ***Alcohol consumption (At least once a week vs. Less than once a week)*** |  |
| Total Effect | 0.42 (0.38-0.46)*** |
| Direct effect | 0.42 (0.38-0.47)*** |
| Indirect effect | 0.99 (0.97-1.00) |
| Percentage mediated (%) | 0.9 (-0.2 to 2.1) |
| ***Tobacco consumption (current smokers vs. No current smokers)*** |  |
| Total Effect | 1.86 (1.64-2.12)*** |
| Direct effect | 1.82 (1.60-2.08)*** |
| Indirect effect | 1.02 (1.00-1.04) |
| Percentage mediated (%) | 4.3 (0.1 to 9.0) |
| **Health status** |  |
| ***Good perceived health vs. Poor perceived health*** |  |
| Total Effect | 0.46 (0.40-0.52)*** |
| Direct effect | 0.49 (0.43-0.56)*** |
| Indirect effect | 0.94 (0.92-0.95)*** |
| Percentage mediated (%) | 5.8 (3.7 to 8.6)*** |
| ***Poor mental health status*** |  |
| Total Effect | 1.46 (1.25-1.70)*** |
| Direct effect | 1.39 (1.19-1.63)** |
| Indirect effect | 1.05 (1.02-1.08)** |
| Percentage mediated (%) | 15.4 (7.7 to 27.6)** |
| **Preventive health care** |  |
| ***Preventive dental visit vs. No preventive dental visit*** |  |
| Total Effect | 0.48 (0.43-0.54)*** |
| Direct effect | 0.49 (0.44-0.54)*** |
| Indirect effect | 0.98 (0.96-0.99)** |
| Percentage mediated (%) | 1.4 (1.1 to 3.2) |

*^a^ Certain health related outcomes were not included because after controlling for confounding factors, the association between these outcomes and education or health literacy was no longer significant; ^b^ Adjusted by age and sex; Bootstrap Percentile 95% Confidence Limits; ** p < 0.05; *** p < 0.0001. All P values are two-tailed.*

*.*

Table A3: Mediation effects of health literacy (reference = sufficient level of health literacy) in the relationship between health related outcomes^a^ and household income (reference = higher household income), HISlink 2018, Belgium

|  | **Odds Ratio^b^**  **(95% CI)** |
| --- | --- |
| **Health behaviour** |  |
| ***Practice of physical activity vs. No practice of physical activity*** |  |
| Total Effect | 0.66 (0.58-0.74)*** |
| Direct effect | 0.68 (0.60-0.77)*** |
| Indirect effect | 0.97 (0.95-0.98)*** |
| Percentage mediated (%) | 6.6 (3.2 to 12.0)** |
| ***Healthy diet vs. Unhealthy diet*** |  |
| Total Effect | 0.77 (0.65-0.89)** |
| Direct effect | 0.79 (0.67-0.92)** |
| Indirect effect | 0.96 (0.94-0.98)** |
| Percentage mediated (%) | 12.1 (5.0 to 33.0)** |
| ***Alcohol consumption (At least once a week vs. Less than once a week)*** |  |
| Total Effect | 0.53 (0.48-0.59)*** |
| Direct effect | 0.54 (0.48-0.60)*** |
| Indirect effect | 0.99 (0.98-1.00) |
| Percentage mediated (%) | 1.3 (-0.2 to 2.9) |
| ***Tobacco consumption (current smokers vs. No current smokers)*** |  |
| Total Effect | 1.73 (1.50-1.99)*** |
| Direct effect | 1.69 (1.46-1.95)*** |
| Indirect effect | 1.02 (1.01-1.04)** |
| Percentage mediated (%) | 4.6 (1.0 to 9.3)** |
| **Health status** |  |
| ***Good self-rated health vs. Poor self-rated health*** |  |
| Total Effect | 0.47 (0.41-0.55)*** |
| Direct effect | 0.50 (0.43-0.57)*** |
| Indirect effect | 0.95 (0.93-0.97)*** |
| Percentage mediated (%) | 4.5 (2.7 to 7.2)*** |
| ***Poor mental health status*** |  |
| Total Effect | 1.57 (1.34-1.84)*** |
| Direct effect | 1.51 (1.29-1.78)*** |
| Indirect effect | 1.04 (1.02-1.07)** |
| Percentage mediated (%) | 10.4 (4.9 to 18.7)** |
| **Preventive health care** |  |
| ***Preventive dental visit vs. No preventive dental visit*** |  |
| Total Effect | 0.57 (0.51-0.64)*** |
| Direct effect | 0.58 (0.52-0.65)*** |
| Indirect effect | 0.98 (0.97-0.99)** |
| Percentage mediated (%) | 2.5 (0.7 to 4.9)** |

*^a^ Certain health related outcomes were not included because after controlling for confounding factors, the association between these outcomes and education or health literacy was no longer significant; ^b^ Adjusted by age and sex; Bootstrap Percentile 95% Confidence Limits; ** p < 0.05; *** p < 0.0001. All P values are two-tailed.*

*.*

**Sensitivity analysis**

**Purchase of antidepressants (90 DDD threshold)**

Table A4: Association between HL and independents variables (mediator model) and mediation effects of HL in the relationship between mental health status and independent variables (summary of effects). Purchase of antidepressants is defined using the 90 DDD threshold per year

|  | **Odds Ratio^a^ (95% CI)** |
| --- | --- |
|  |  |
| **Mediator model: *Insufficient level of HL vs. Sufficient level of HL*** | |
| **Educational attainment** |  |
| Higher secondary education or lower | 2.19 (1.91-2.50)*** |
| Higher education | 1 |
| **Household income category** |  |
| Lower income | 1.45 (1.29-1.62)*** |
| Higher income | 1 |
| **Summary of effects of educational attainment** |  |
| Total Effect | 1.00 (0.79-1.21) |
| Direct effect | 0.94 (0.74-1.15) |
| Indirect effect | 1.06 (0.99-1.14) |
| Percentage mediated (%) | 3008 (-325412 to 331427) |
| **Summary of effects of household income** |  |
| Total Effect | 1.45 (1.19-1.72)** |
| Direct effect | 1.40 (1.14-1.65)** |
| Indirect effect | 1.04 (1.02-1.07)** |
| Percentage mediated (%) | 12.7 (4.0 to 21.3)** |

*. ^a^ Adjusted by age and sex; ** p < 0.05; *** p < 0.0001.*
